# Financial toxicity and psychological distress in cardiovascular non-cancer multimorbidity: a latent profile analysis and causal weighting

**DOI:** 10.3389/fpsyg.2025.1633445

**Published:** 2025-08-15

**Authors:** Ruihan Wu, Jiali Zhou, Huafen Liu, Shanshan Liu, Xiaojie Ma, Tian Li, Jingjing Tao, Zhongxiang Cai

**Affiliations:** ^1^Department of Nursing, Renmin Hospital of Wuhan University, Wuhan, China; ^2^Department of Cardiology, Renmin Hospital of Wuhan University, Wuhan, China; ^3^Department of Geriatrics, Renmin Hospital of Wuhan University, Wuhan, China

**Keywords:** cardiovascular, non-cancer multimorbidity, financial toxicity, anxiety, depression, latent profile analysis, inverse probability of treatment weighting, doubly robust estimation

## Abstract

**Background:**

Cardiovascular disease with multimorbidity imposes substantial long-term financial burdens. Financial toxicity, the financial burden associated with healthcare, is increasingly recognized as a psychosocial stressor affecting health outcomes among chronically ill populations. However, its psychological heterogeneity in non-cancer patients is understudied. We aimed to identify financial toxicity profiles, their determinants, and associations with anxiety/depression in cardiovascular non-cancer multimorbidity.

**Methods:**

This study used a cross-sectional design. Utilizing latent profile analysis to identify distinct financial toxicity subgroups. Univariate and multivariate logistic regression analyses were employed to explore factors associated with subgroup membership. Inverse probability of treatment weighting based on covariate balancing propensity scores was applied to assess the associations between financial toxicity profiles and psychological distress. Doubly robust regression models were utilized to validate the robustness of the observed associations.

**Results:**

Financial toxicity was categorized into three latent profiles: mild (63.8%), moderate (11.0%), and none (25.2%). Demographic, economic, social support, and coping variables significantly differed across profiles. In weighted regression models using inverse probability of treatment weighting based on covariate balancing propensity scores, both mild and moderate toxicity were associated with elevated anxiety (*β* = 1.88 and 8.68, respectively) and depression scores (*β* = 4.21 and 11.92; all *p* < 0.01) compared to the no-toxicity group. These associations remained robust in doubly robust models adjusting for covariates (GAD-7: *β* = 1.72 and 8.31; PHQ-9: *β* = 4.08 and 11.48).

**Conclusion:**

Financial toxicity is prevalent and heterogeneous in cardiovascular multimorbidity. Its distinct profiles predict elevated psychological distress, supporting targeted interventions to alleviate financial burden and enhance mental health resilience.

## Introduction

Chronic non-communicable diseases (NCDs) are a major global health burden due to their chronicity and complexity. Among them, cardiovascular diseases (CVDs) are leading in morbidity, mortality, and treatment costs, posing serious challenges to public health and health systems (Ferrari et al., 2024; Kandula et al., 2024). Patients with CVDs often suffer from multiple chronic conditions such as diabetes or chronic kidney disease, forming a multimorbidity state centered on cardiovascular dysfunction (Bullock et al., 2011). This inevitably leads to long-term care needs, frequent hospitalizations, and escalated costs, thereby inducing psychological distress (e.g., anxiety and depression) (Ren et al., 2025). These factors collectively exacerbate the risks of treatment discontinuation and poor outcomes.

This economic burden, often referred to as financial toxicity (FT), includes both the objective financial cost and the subjective stress experienced by patients and families due to high out-of-pocket spending (Zafar and Abernethy, 2013; Valero-Elizondo et al., 2021). Although initially described in cancer care, the concept of financial toxicity is increasingly recognized in chronic disease contexts, particularly in CVDs, where it reflects a growing concern for health equity and sustainable disease management.

While FT has been widely studied in cancer care, emerging evidence suggests that patients with cardiovascular disease may experience equal or even greater financial burden, due to the chronic, progressive nature of CVDs and the frequent coexistence of multiple conditions such as diabetes and kidney disease (Valero-Elizondo et al., 2021). However, this burden in CVDs populations remains underrecognized and underexplored in research and policy (Valero-Elizondo et al., 2021). Given the growing prevalence of multimorbidity and rising healthcare costs, there is an urgent need to assess and address FT among individuals with CVDs.

In recent years, FT has become a key issue in chronic disease research. While early studies mainly focused on cancer (Ferrari et al., 2024; Kandula et al., 2024), more attention is now given to cardiovascular and cerebrovascular diseases (Bullock et al., 2011). Patients often face high medical costs due to long-term treatment and repeat hospital visits. Studies show that 16 to 73% of patients with chronic illnesses may experience FT (Kandula et al., 2024; Zafar and Abernethy, 2013). FT can lead to emotional stress, anxiety, depression, and lower quality of life (Valero-Elizondo et al., 2021). It may also cause patients to delay or stop treatment, which can result in worse health and higher death rates.

To better understand FT, some studies use Latent Profile Analysis (LPA). This method helps identify groups of patients who share similar financial stress patterns (Roth et al., 2020). Research has found that FT can be grouped into different levels, such as low, medium, and high (Zafar and Abernethy, 2013; Violan et al., 2014). These groupings reflect differences in financial burden and coping capacity. Since treatment needs, disease progression, and medical costs vary across diseases, FT patterns may differ by condition (Bullock et al., 2011). Moreover, to further explore the associations between different FT profiles and psychological outcomes such as anxiety and depression, inverse probability of treatment weighting (IPTW) based on covariate balancing propensity scores (CBPS) was applied to adjust for baseline differences and improve covariate balance across groups (Chesnaye et al., 2021). In addition, doubly robust (DR) regression models—combining IPTW with multivariable covariate adjustment—were used to generate more reliable estimates and account for potential residual confounding.

## Methods

### Study design and participants

A convenience sampling method was used to recruit 345 patients with cardiovascular non-cancer chronic comorbidities from the Department of Cardiology at a tertiary hospital in Wuhan Province between November 2024 and January 2025. The inclusion criteria were as follows: (1) age ≥18 years; (2) diagnosis of ≥2 chronic diseases, including at least one cardiovascular condition, confirmed by a secondary or higher-level hospital, with the first diagnosis made ≥3 months prior to enrollment; (3) New York Heart Association (NYHA) functional class II or III; (4) ability to communicate effectively and complete the questionnaire independently or with assistance as desired; (5) clinically stable condition.

Exclusion criteria included: (1) diagnosis of malignant tumors; (2) acute-phase diseases (e.g., acute myocardial infarction, acute heart failure), unstable clinical condition, or serious complications; (3) visual, auditory, cognitive, or speech impairments; (4) withdrawal from the study before completion.

### Sample size

Logistic regression analysis requires the sample size to be 10–15 times of the number of independent variables, this study contains 22 independent variables, the sample size is 220–330 cases, and then considering the 20% lost visit rate, the sample size should be 275–413 cases. A total of 360 questionnaires were distributed in this study, and 345 valid questionnaires were recovered, of which 22 were filled in by the researcher, and the validity rate of the questionnaires was 95.8%. This study was reviewed by the Ethics Committee of Renmin Hospital of Wuhan University (WDRY2024-K277).

### Data collection

Questionnaires were distributed after obtaining informed consent from the patients. They were completed by the patients themselves; for those with difficulty completing the questionnaires, the researcher assisted by filling them out based on the patients’ verbal responses after reviewing the content with them. All questionnaires were administered and collected on-site by the researcher. A standardized instruction was provided to explain the items and the method of completion. The questionnaire was designed to be completed within 15–30 min. Upon completion, the researcher checked each questionnaire on-site to ensure completeness. Missing responses were promptly addressed, and any unclear entries were verified with the participants. Questionnaires with unresolved missing or unclear items within the allotted time were deemed invalid.

### Research tools

#### General information questionnaire

This tool was developed by the researcher based on a comprehensive literature review. Some information was also obtained from hospitalization records and verified with patients during the survey. Collected data included general demographic characteristics (gender, age, place of residence, education level, marital status, occupation, type of health insurance, and per capita monthly household income) as well as disease-related variables (number and types of chronic comorbidities, duration since the initial diagnosis, average annual relapse frequency, and patients’ level of awareness regarding treatment costs).

#### Comprehensive scores for financial toxicity based on the patient-reported outcome measures (COST-PROM)

The scale, developed by De Souza et al. (2014), was the first tool designed to assess financial toxicity among cancer patients. It consists of 11 items across three dimensions: financial burden, available economic resources, and psychosocial responses. The Chinese version was translated and validated by Yu (Yu et al., 2021), demonstrating good internal consistency with a Cronbach’s *α* of 0.889, and has been widely applied among cancer patients in China. In 2021, Pavela et al. (2021) further confirmed the scale’s internal consistency and validity in a sample of patients with chronic diseases, supporting its utility as a screening tool for financial toxicity in non-cancer chronic conditions. In our study, internal consistency was high (Cronbach’s α = 0.944). Item-total correlations ranged from 0.627–0.882, indicating strong item homogeneity. The scale uses a 5-point Likert scale (0 to 4) with total scores ranging from 0 to 44. Scores ≥26 indicate no FT, 14–25 mild FT, 1–13 moderate FT, and 0 severe FT.

#### Social support rating scale (SSRS)

Developed by Professor Xiao (1994) in 1986, this scale is used to assess an individual’s level of social support. It comprises three dimensions: subjective support (4 items), objective support (3 items), and support utilization (3 items), with a total of 10 items. The total score ranges from 12 to 66, with higher scores indicating greater levels of social support. The scale demonstrates good internal consistency, with a Cronbach’s alpha of 0.89. The evaluation criteria are as follows: scores of 12–22 indicate low social support, 23–44 indicate moderate support, and 45–66 indicate high support.

#### Medical coping modes questionnaire (MCMQ)

Originally developed by Feifel et al. (1987) and later translated and adapted into Chinese by Shen and Jiang (2000), the scale assesses coping styles across three dimensions: confrontation (8 items), avoidance (7 items), and submission (5 items), totaling 20 items. It uses a 4-point Likert scale, with responses ranging from 1 to 4 based on the frequency of each coping behavior, yielding a total score between 20 and 80. Higher scores in each dimension indicate a stronger tendency toward that particular coping style. The scale has demonstrated good reliability and validity and is primarily used among cancer and chronic disease patients in China.

#### Generalized anxiety disorder-7 (GAD-7)

The scale was developed by Spitzer et al. (2006) in 2006 to assess the anxiety symptoms of an individual within the past 2 weeks with good reliability and validity. The scale consists of seven entries, each of which is scored according to the frequency of symptoms: 0 (not at all), 1 (for a few days), 2 (for more than half of the days), and 3 (almost every day). The total score ranges from 0 to 21, with higher scores indicating more severe anxiety. 0 to 4 indicates no or minimal anxiety, 5 to 9 indicates mild anxiety, 10 to 14 indicates moderate anxiety, and 15 to 21 indicates severe anxiety.

#### Patient health questionnaire-9 (PHQ-9)

This is a self-assessment scale developed by Kroenke et al. (2001) in 1999 to help identify and assess an individual’s depressive symptoms in the past 2 weeks. The scale consists of nine entries, each of which is scored according to the frequency of symptoms: 0 (not at all), 1 (a few days), 2 (more than half the days), and 3 (almost every day). The total score ranges from 0 to 27, with higher scores indicating more severe depression. National and international studies have confirmed that the PHQ-9 has good reliability and validity, and the American College of Cardiology recommends it as a screening tool for depression in patients with cardiovascular disease (Carney et al., 2005).

### Statistical analysis

SPSS 27.0 was used for data analysis and processing. Measurement data conforming to a normal distribution were expressed as mean ± standard deviation (x ± s), while categorical variables were described using frequencies and percentages. Differences in general demographic characteristics were assessed using t-tests and one-way ANOVA. LPA was performed using Mplus 8.3. The scores of the 11 items from the Comprehensive Scores for Financial Toxicity Based on the Patient-Reported Outcome Measures, COST-PROM were used as observed indicators, and models with one to five latent profiles were sequentially estimated. Model fit was evaluated using the following criteria: (1) Information criteria: Akaike Information Criterion (AIC), Bayesian Information Criterion (BIC), and adjusted BIC (aBIC) were used to assess model fit, with lower values indicating better fit. (2) Entropy: This index measures classification accuracy, ranging from 0 to 1. Higher values indicate better classification, with values ≥0.8 suggesting over 90% classification accuracy, while values ≤0.6 suggest error rates above 20%. (3) Likelihood ratio tests: These included the Lo–Mendell–Rubin adjusted likelihood ratio test (LMR) and the Bootstrap likelihood ratio test (BLRT), which compare models with k versus k–1 classes. A significant *p*-value (*p* < 0.05) indicates that the k-class model fits better than the k–1 class model. Model selection was based on statistical indicators and interpretability.

LPA was conducted to classify participants into three distinct financial toxicity profiles (Berlin et al., 2013). After LPA, inverse probability of treatment weighting (IPTW) based on covariate balancing propensity scores (CBPS) was applied to address potential confounding across the profiles. CBPS was selected because it directly incorporates covariate balance into the propensity score estimation process, improving robustness compared to conventional approaches like nearest neighbor or kernel matching, which may discard unmatched cases or require bandwidth tuning, respectively (Imai and Ratkovic, 2014). Propensity scores were estimated using logistic regression models, with variables that were statistically significant in univariate analysis used as covariates. Stabilized weights were then applied in weighted linear regression models to evaluate differences in anxiety and depression scores across the profiles.

Covariate balance before and after weighting was assessed using standardized mean differences (SMDs), with values < 0.1 indicating good balance. Love plots were generated to visualize covariate alignment. While most variables achieved acceptable balance, some showed residual imbalance.

**Figure 1:**
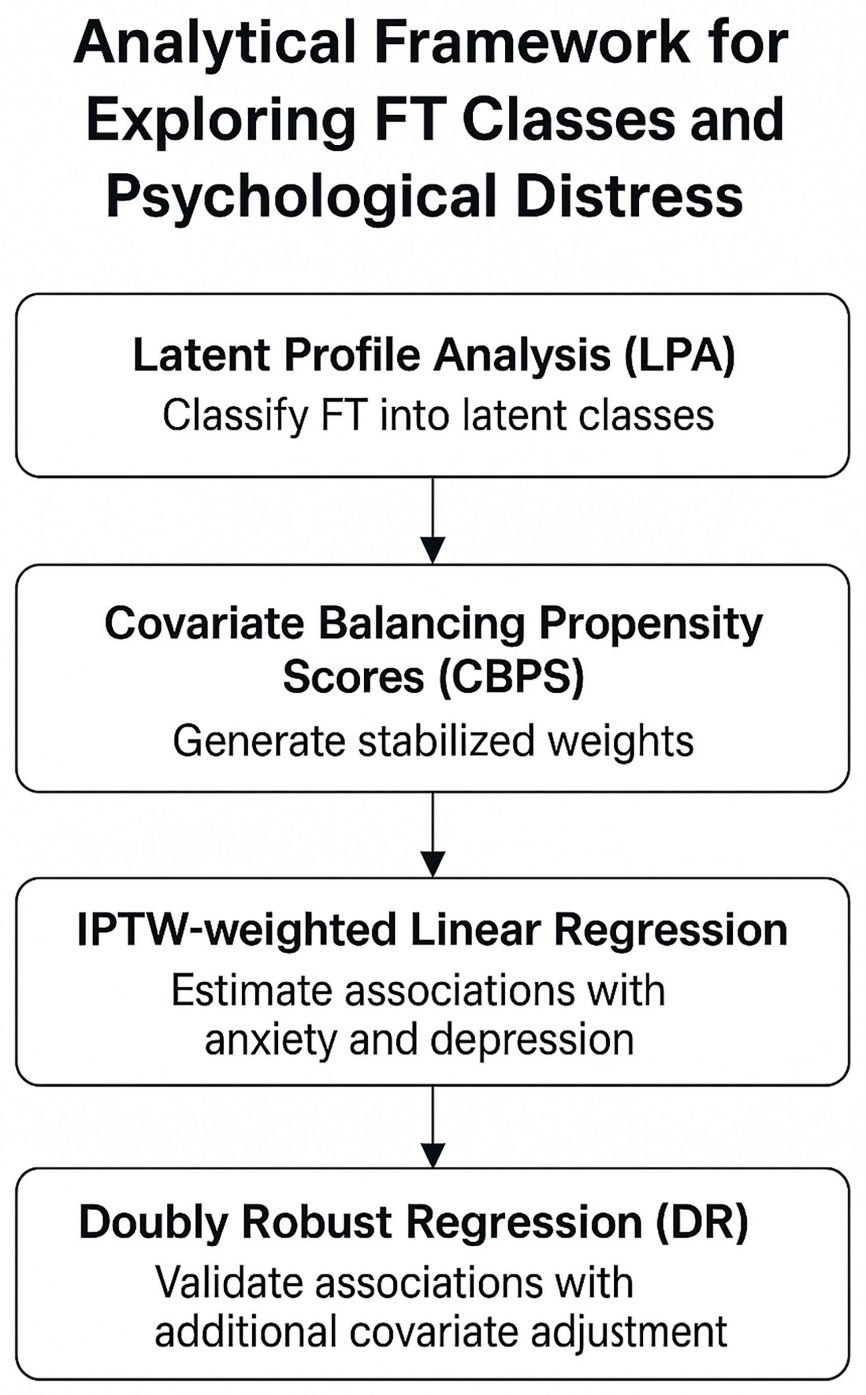
Analytical framework outlining the stepwise approach used to examine the relationship between financial toxicity (FT) and psychological distress. Latent profile analysis (LPA) identified FT subgroups, followed by covariate balancing propensity scores (CBPS) to generate stabilized weights. Inverse probability of treatment weighting (IPTW) – weighted regression and doubly robust (DR) models were then used to estimate and validate associations.

To ensure robustness, doubly robust regression models were conducted by incorporating the imbalanced covariates into the outcome models alongside the IPTW weights. The analytical framework is detailed in Figure 1. Results consistently demonstrated significant differences in psychological distress across profiles, reinforcing the validity of the observed associations between financial toxicity and mental health outcomes. All CBPS estimation, IPTW modeling, and doubly robust analyses were performed using R version 4.5.0.

## Results

### Participant characteristics

A total of 345 patients with cardiovascular non-cancer chronic comorbidities were included, comprising 192 males and 153 females. The age distribution was 42 (18–45 years), 111 (46–65 years), and 192 (≥65 years). Most participants lived in urban areas (*n* = 256), with 89 from rural areas. Educational levels were primary or below (*n* = 125), secondary (*n* = 151), and college or above (*n* = 69). There were 284 married and 61 unmarried participants. Occupations included farmers (*n* = 66), workers (*n* = 38), institutional staff (*n* = 24), retirees (*n* = 106), and others (*n* = 111). Health insurance types included urban resident (*n* = 175), urban employee (*n* = 149), rural cooperative (*n* = 14), and self-paid (*n* = 7). Monthly household income per capita was ≤3,000 yuan (*n* = 170), 3,001–5,000 yuan (*n* = 119), and ≥5,000 yuan (*n* = 56). Regarding disease burden, 137 had two chronic conditions, 174 had 3–5, and 34 had more than five. Time since first diagnosis was ≤1 year (*n* = 24), 1–5 years (*n* = 79), and >5 years (*n* = 242). Annual relapse frequency was ≤1 (*n* = 171), 1–5 (*n* = 142), and >5 (*n* = 32). Treatment cost awareness was classified as none (*n* = 78), partial (*n* = 208), and full (*n* = 59).

### Identification of financial toxicity subgroups

**Figure 2:**
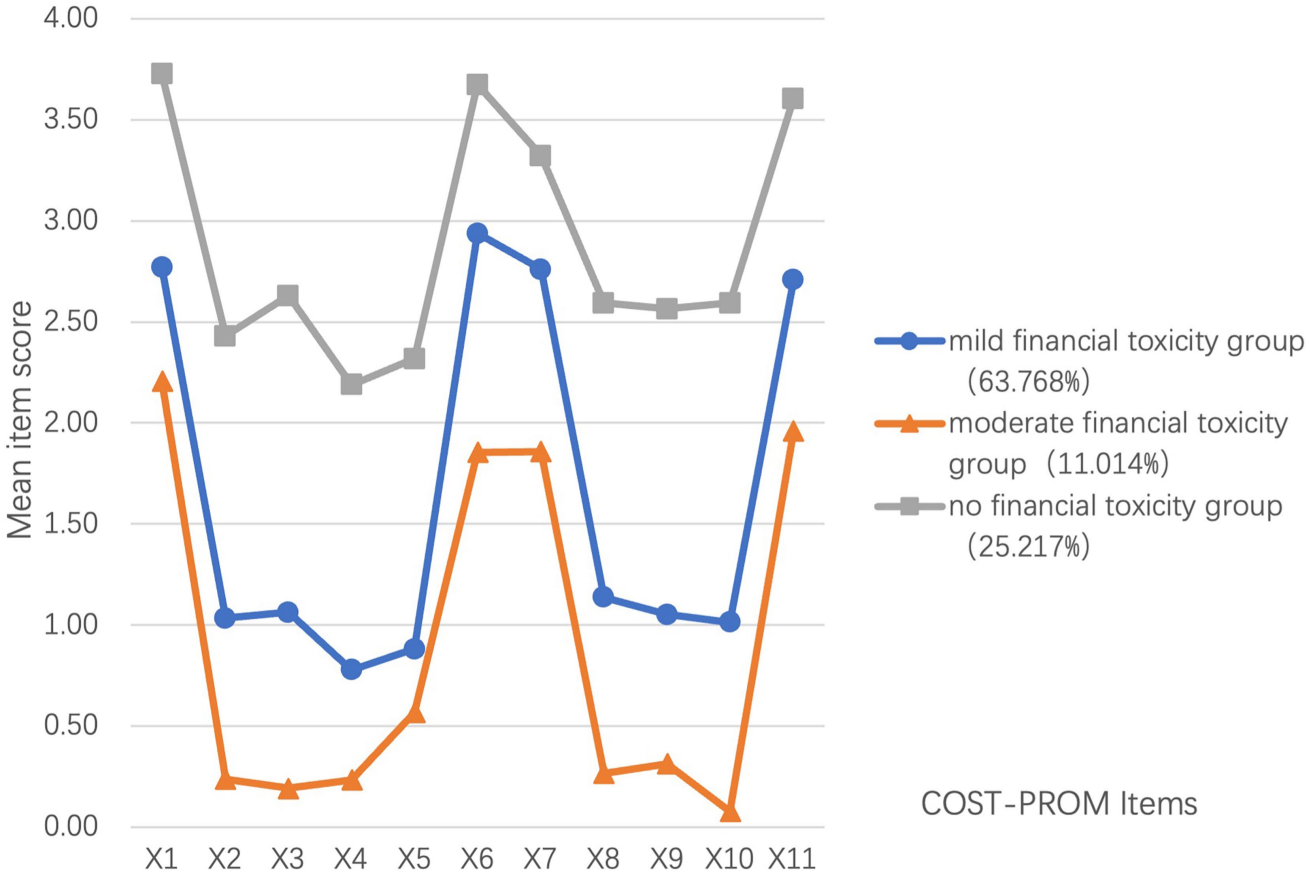
Mean item-level scores across 11 COST-PROM items for the three latent financial toxicity (FT) profiles identified through latent profile analysis. Lower scores reflect greater financial burden. The moderate FT group (11.0%) consistently scored lowest on most items, followed by the mild FT group (63.8%) and the no FT group (25.2%). This pattern illustrates distinct perceived financial stress across profiles.

A total of five latent profile models were fitted, as shown in Table 1. With the increase in the number of classes, the AIC, BIC, and aBIC values gradually decreased. The LMR and BLRT tests were statistically significant (*p* < 0.05) for the 2- and 3-class models. Entropy values were highest and equal for the 2- and 4-class models. However, LMR was no longer significant when the number of classes increased to four or five. So, the 3-class model is relatively appropriate choice. Beyond statistical fit, the three-class model was chosen for its clinical relevance and interpretability. It offers a simple, actionable framework aligning with existing clinical frameworks that healthcare providers can adopt in routine care. It can help healthcare providers identify at-risk patients and offer personalized support. Considering both statistical indicators and clinical interpretability, the 3-class model was selected as the optimal solution. Three latent profiles based on the 11 observed variables were identified (see Figure 2). The mean scores for each profile were 18.123, 9.605, and 31.655. Based on the FT scoring criteria (≥26: no FT; 14–25: mild; 1–13: moderate), the profiles were labeled as no FT group, mild FT group, and moderate FT group, respectively.

**Table 1:** Model fit indices for the latent profile analysis of financial toxicity in patients with cardiovascular non-cancer multimorbidity.

Model	AIC	BIC	aBIC	Entropy	LMR (P)	BLRT (P)	Number of classes (*n*)	Posterior class probability (%)
1	9646.594	9731.152	9661.362	–	–	–	–	–
2	7543.223	7673.903	7566.046	0.983	<0.001	<0.001	254/91	73.623/26.377
3	7095.396	7272.199	7126.274	0.969	0.0205	<0.001	220/38/87	63.768/11.014/25.217
4	6611.211	6834.137	6650.145	0.983	0.1228	<0.001	35/64/203/43	10.145/18.551/58.841/12.464
5	6508.562	6777.611	6555.551	0.939	0.1013	<0.001	32/58/149/63/43	9.275/16.812/43.188/18.261/12.464

### Univariate analysis of latent profiles of financial toxicity

Univariate analysis showed no statistically significant differences (*p* > 0.05) among the latent profiles of financial toxicity with respect to gender and scores on the confrontation and submission dimensions of medical coping. However, significant differences (*p* < 0.05) were observed across the profiles in relation to age, place of residence, education, marital status, occupation, type of health insurance, per capita monthly household income, number of chronic comorbidities, duration since first diagnosis, frequency of annual disease recurrence, awareness of treatment costs, types of comorbid chronic diseases (including chronic heart failure, arrhythmia, COPD, chronic bronchitis, chronic gastritis, chronic hepatitis, other digestive disorders, chronic renal failure, stroke, and cervical spondylosis), total and subscale scores of social support (objective support, subjective support, and support utilization), total medical coping score, and the avoidance dimension of coping style. Only variables with statistically significant differences are presented in Tables 2, 3.

**Table 2:** Univariate analysis of general characteristics and latent profiles of financial toxicity among patients with cardiovascular non-cancer multimorbidity [*N* = 345, *n*(%)].

Variable	Mild financial toxicity (*n* = 220)	Moderate financial toxicity (*n* = 38)	No financial toxicity (*n* = 87)	*χ*^2^or*F*	*p*-value
Age				24.424	<0.001
18_45 years (inclusive)	23 (10.5)	7 (18.4)	12 (13.8)		
45_65 years	78 (35.5)	20 (52.6)	13 (14.9)		
65 years and above	119 (54.1)	11 (28.9)	62 (71.3)		
Place of residence				14.066	0.001
Urban	171 (77.7)	33 (86.8)	52 (59.8)		
Rural	49 (22.3)	5 (13.2)	35 (40.2)		
Education Level				78.189	<0.001
Primary school or below			54 (62.1)		
Middle to high school			24 (27.6)		
College and above			9 (10.3)		
Marital Status				11.876	0.003
Married	191 (86.8)	30 (78.9)	61 (70.1)		
Unmarried (never married, divorced, or widowed)	29 (13.2)	8 (21.1)	26 (29.9)		
Occupation				48.765	<0.001
Worker	28 (12.7)	8 (21.1)	2 (2.3)		
Farmer	34 (15.5)	1 (2.6)	31 (35.6)		
Public sector employee	14 (6.4)	7 (18.4)	3 (3.4)		
Retired	80 (36.4)	11 (28.9)	15 (17.2)		
Type of health insurance				65.582	<0.001
Urban employee insurance		34 (89.5)	13 (14.9)		
Resident basic health insurance		3 (7.9)	70 (80.5)		
Self-paying		1 (2.6)	4 (4.6)		
Monthly household income *Per Capita*				85.345	<0.001
≤3,000 RMB	98 (44.5)	1 (2.6)	71 (81.6)		
3,000–5,000 RMB	87 (39.5)	18 (47.4)	14 (16.1)		
≥5,000 RMB	35 (15.9)	19 (50.0)	2 (2.3)		
Number of chronic comorbidities				25.738	<0.001
2 types	94 (42.7)	24 (63.2)	19 (21.8)		
3–5 types	109 (49.5)	13 (34.2)	52 (59.8)		
≥5types	17 (7.7)	1 (2.6)	16 (18.4)		
Years since first chronic disease diagnosis				15.186	0.004
≤1 year	13 (5.9)	5 (13.2)	6 (6.9)		
1–5 years	57 (25.9)	13 (34.2)	9 (10.3)		
≥5 years	150 (68.2)	20 (52.6)	72 (82.8)		
Average annual recurrence of chronic disease				30.609	<0.001
≤1 time	120 (54.5)	28 (73.7)	23 (26.4)		
1–5 times	81 (36.8)	7 (18.4)	54 (62.1)		
≥5 times	19 (8.6)	3 (7.9)	10 (11.5)		
Awareness of treatment costs				70.156	<0.001
Completely unaware	27 (12.3)	4 (10.5)	47 (54.0)		
Partially aware	149 (67.7)	23 (60.5)	36 (41.4)		
Fully aware	44 (20.0)	11 (28.9)	4 (4.6)		
Types of comorbid chronic diseases					
Chronic heart failure – Yes			19 (21.8)	23.725	<0.001
Chronic heart failure – No			68 (78.2)		
Arrhythmia – Yes			17 (19.5)	6.025	0.049
Arrhythmia – No			70 (80.5)		
COPD – Yes			20 (23.0)	9.163	0.01
COPD – No			67 (77.0)		
Chronic bronchitis – Yes			17 (19.5)	7.888	0.019
Chronic bronchitis – No			70 (80.5)		
Chronic gastritis – Yes			30 (34.5)	6.971	0.031
Chronic gastritis – No			57 (65.5)		
Chronic hepatitis – No			78 (89.7)		
Other digestive diseases – No			79 (90.8)		
Chronic kidney failure – Yes			11 (12.6)	22.182	<0.001
Chronic kidney failure – No			76 (87.4)		
Stroke – Yes			19 (21.8)	16.374	<0.001
Stroke – No			68 (78.2)		
Cervical spondylosis – Yes			2 (2.3)	6.102	0.047
Cervical spondylosis – No			85 (97.7)		

**Table 3:** Comparison of social support and medical coping scores across latent profiles of financial toxicity in patients with cardiovascular non-cancer multimorbidity [*n* = 345, mean ± SD].

Variable	Mild financial toxicity (*n* = 220)	Moderate financial toxicity (*n* = 38)	No financial toxicity (*n* = 87)	*χ*^2^ or *F*	*p*-value
Total score of SSRS	39.39 ± 7.010	43.95 ± 6.592	31.06 ± 5.518	75.016	<0.001
Objective support dimension	10.15 ± 3.065	11.71 ± 3.279	7.72 ± 2.424	31.020	<0.001
Subjective support dimension	21.14 ± 4.282	23.13 ± 3.905	16.52 ± 3.125	61.828	<0.001
Support utilization	8.09 ± 1.440	9.11 ± 1.351	6.80 ± 1.256	43.553	<0.001
Total Score of MCMQ	50.68 ± 3.557	51.21 ± 4.028	49.49 ± 3.707	4.236	0.015
Avoidance dimension	17.43 ± 1.911	18.92 ± 2.259	16.66 ± 2.096	17.065	<0.001

### Multivariate logistic regression analysis

Multivariate logistic regression was conducted using the three latent profiles of financial toxicity among patients with cardiovascular non-cancer chronic comorbidities as the dependent variable. Independent variables included factors found to be statistically significant in the univariate analysis. The results indicated that age, place of residence, educational level, marital status, occupation, type of health insurance, per capita monthly household income, years since first diagnosis, average annual recurrence of chronic disease, social support (subjective support and support utilization), and medical coping style (avoidance dimension) were significantly associated with the different financial toxicity profiles (all *p* < 0.05), as shown in Table 4.

**Table 4:** Logistic regression of influencing factors for financial toxicity profiles.

Independent variables	Mild financial toxicity group^a^					Moderate financial toxicity group^a^				
	B	OR	Wald χ^2^	95%CI	*p*	B	OR	Wald χ^2^	95%CI	*p*-value
Intercept	−8.564		12.158		<0.001	−23.444		21.254		<0.001
Age	−2.844	0.058	5.986	0.006 ~ 0.568	0.014	−3.303	0.037	3.452	0.001 ~ 1.199	0.063
18–45 years										
Place of Residence	−0.321	0.726	0.451	0.284 ~ 1.851	0.502	−2.356	0.095	5.256	0.013 ~ 0.710	0.022
Urban										
Education: Middle to High School	−1.545	0.213	2.193	0.028 ~ 1.648	0.139	−3.296	0.037	6.325	0.003 ~ 0.483	0.012
Marital Status: Unmarried	0.713	2.04	1.519	0.657 ~ 6.342	0.218	2.979	19.677	8.154	2.546 ~ 152.097	0.004
Occupation: Retired	1.468	4.34	5.72	1.303 ~ 14.452	0.017	−0.002	0.998	0.000	0.097 ~ 10.305	0.999
Health Insurance Type: Self-paying	−3.983	0.019	5.512	0.001 ~ 0.518	0.019	−3.585	0.028	2.106	0.000 ~ 3.516	0.147
Monthly Household Income ≤3,000 RMB	−0.737	0.478	0.92	0.106 ~ 2.158	0.338	−5.182	0.006	8.122	0.000 ~ 0.198	0.004
Monthly Household Income ≥5,000 RMB	1.336	3.803	1.686	0.506 ~ 28.556	0.194	3.241	25.557	6.460	2.100 ~ 311.059	0.011
Number of Chronic Comorbidities ≥5	−2.363	0.094	9.078	0.020 ~ 0.438	0.003	−3.612	0.027	5.080	0.001 ~ 0.624	0.024
Number of Chronic Comorbidities 3–5	−1.503	0.222	7.421	0.075 ~ 0.656	0.006	−2.368	0.094	8.097	0.018 ~ 0.479	0.004
Years Since First Chronic Disease Diagnosis ≤1	−4.435	0.012	9.913	0.001 ~ 0.187	0.002	−4.871	0.008	5.826	0.000 ~ 0.400	0.016
Annual Recurrence of Chronic Disease ≤1	1.976	7.211	6.163	1.516 ~ 34.305	0.013	1.450	4.265	1.158	0.304 ~ 59.848	0.282
Subjective Support	0.384	1.467	15.713	1.214 ~ 1.774	<0.001	0.506	1.659	12.645	1.255 ~ 2.193	<0.001
Support Utilization	0.52	1.683	7.483	1.159 ~ 2.443	0.006	0.944	2.571	11.268	1.481 ~ 4.463	0.001
Medical Coping Modes: Avoidance Dimension	0.018	1.018	0.028	0.828 ~ 1.252	0.868	0.666	1.945	11.501	1.324 ~ 2.858	0.001

a: Both groups above use the no financial toxicity group as the reference.

### Balance assessment

**Figure 3:**
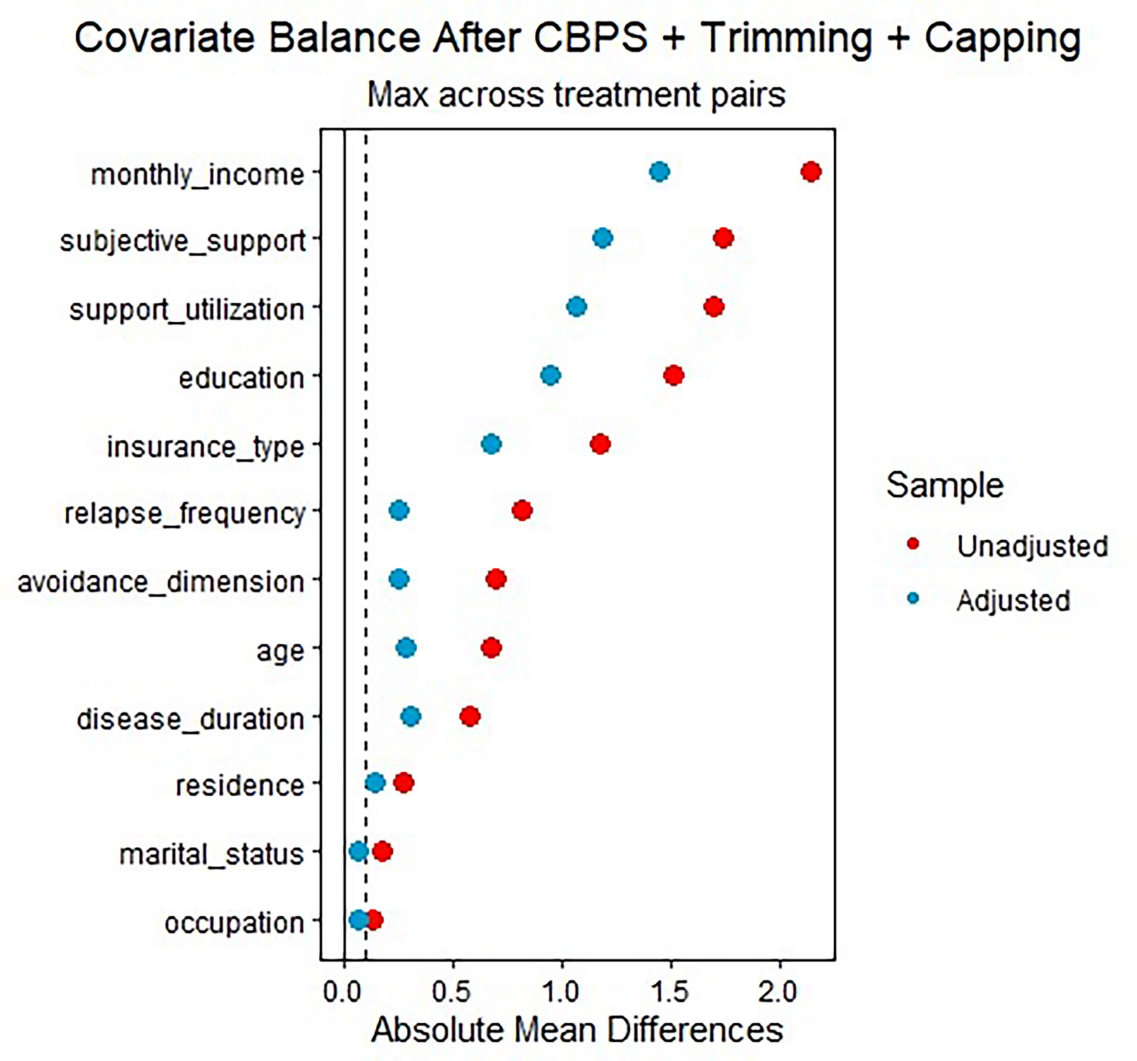
Standardized mean differences (SMDs) for baseline covariates before (red) and after (blue) weighting using covariate balancing propensity scores (CBPS), with 5% trimming and a maximum cap of 10. The plot displays the maximum absolute SMD across financial toxicity profile pairs. The vertical dashed line at 0.1 indicates the commonly accepted threshold for acceptable covariate balance. Weighting improved overall balance, although residual imbalance remained in variables such as monthly income and subjective support.

After applying IPTW based on CBPS, covariate balance across financial toxicity profiles improved substantially. As shown in Figure 3, standardized mean differences (SMDs) for most covariates were reduced, with many falling below the 0.1 threshold. However, residual imbalance persisted for variables such as marital status, occupation, and income, likely due to substantial baseline differences that could not be fully resolved by weighting.

### Weight diagnostics

**Figure 4:**
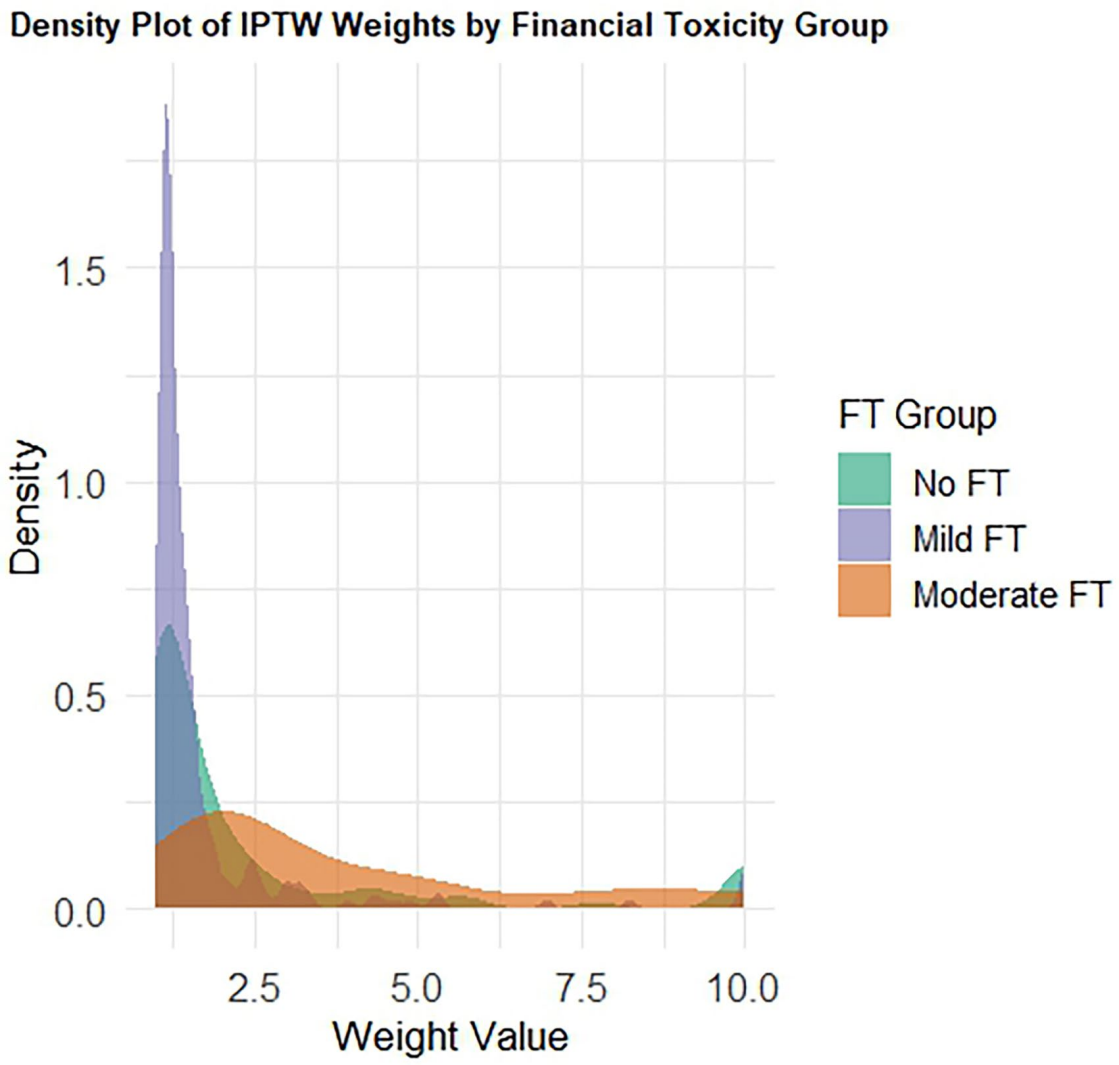
Density plot of stabilized inverse probability of treatment weights (IPTWs) by financial toxicity profile. Weights were estimated using covariate balancing propensity scores (CBPS), with 5% trimming and capping at 10 to address extreme values. Most weights clustered below 2.5, but the moderate FT group displayed a pronounced right-skewed tail, indicating the presence of outlier weights that were subsequently trimmed or capped to stabilize the model.

The distribution of IPTW weights is shown in Figure 4. Most weights were below 2.5, indicating acceptable influence from individual observations. A right-skewed distribution was observed, particularly in the moderate FT group, suggesting the presence of extreme weights. To mitigate the influence of extreme weights and improve model stability, we applied 5% trimming and capped weights at a maximum value of 10. These thresholds were selected based on visual inspection of the weight distribution (Figure 4) and recommendations in prior IPTW literature (Chesnaye et al., 2021). They were also tailored to the specific characteristics of our data, ensuring that extreme values were adequately addressed while retaining a meaningful sample for the analysis. Specifically, trimming extreme weights improves the efficiency of estimates and reduces the variance inflation commonly seen in small subgroups, especially when baseline imbalance is substantial. Capping at 10 ensures that no single observation exerts undue influence on outcome estimates.

### Association between FT and psychological outcomes

Weighted linear regression analyses based on inverse probability of treatment weighting (IPTW) estimated via covariate balancing propensity scores (CBPS) demonstrated significant associations between financial toxicity and psychological outcomes (Tables 5, 6). Compared to participants without financial toxicity, those in the moderate group reported markedly higher GAD-7 (*β* = 8.68, *p* < 0.001) and PHQ-9 scores (*β* = 11.92, *p* < 0.001), while participants in the mild group also showed elevated levels of anxiety (*β* = 1.88, *p* = 0.004) and depression (*β* = 4.21, *p* < 0.001).

**Table 5:** Weighted linear regression results for anxiety (IPTW-adjusted).

Variable	Estimate	Std. Error	t value	*p* value
(Intercept)	5.24	3.22	1.63	0.105
Moderate FT (vs. No FT)	8.68	1.13	7.68	<0.001
Mild FT (vs. No FT)	1.88	0.65	2.91	0.004
Age	0.97	0.68	1.42	0.157
Residence	−0.45	0.49	−0.91	0.366
Education	0.71	0.64	1.11	0.267
Marital status	0.95	0.67	1.41	0.158
Occupation	0.07	0.16	0.42	0.672
Insurance type	0.15	0.50	0.30	0.766
Monthly income	0.64	0.44	1.46	0.146
Disease duration	−1.05	0.53	−1.98	0.049
Relapse frequency	0.86	0.49	1.75	0.082
Subjective support	−0.05	0.06	−0.84	0.404
Avoidance dimension	−0.12	0.12	−1.07	0.286

Weighted linear regression was performed using stabilized inverse probability of treatment weights (IPTW) estimated via covariate balancing propensity scores (CBPS), with 5% trimming and a maximum cap of 10. The dependent variable was GAD-7 anxiety score, and the reference group was participants with no financial toxicity.

**Table 6:** Weighted linear regression results for depression (IPTW-adjusted).

Variable	Estimate	Std. Error	t value	*p* value
(Intercept)	9.99	3.40	2.94	0.0036
Moderate FT (vs. No FT)	11.92	1.10	10.84	<0.001
Mild FT (vs. No FT)	4.21	0.68	6.21	<0.001
Age	1.31	0.63	2.08	0.038
Residence	−0.99	0.53	−1.88	0.061
Education	0.68	0.64	1.06	0.289
Marital status	1.54	0.74	2.08	0.038
Occupation	0.13	0.19	0.69	0.491
Insurance type	0.54	0.56	0.96	0.336
Monthly income	0.44	0.58	0.76	0.447
Disease duration	−1.03	0.54	−1.92	0.056
Relapse frequency	0.70	0.57	1.24	0.215
Subjective support	−0.24	0.08	−3.10	0.002
Support utilization	−0.61	0.27	−2.25	0.025
Avoidance dimension	−0.21	0.14	−1.55	0.122

Weighted linear regression was performed using stabilized inverse probability of treatment weights (IPTW) estimated via covariate balancing propensity scores (CBPS), with 5% trimming and a maximum cap of 10. The dependent variable was PHQ-9 depression score. The no financial toxicity group served as the reference.

In addition, older age (*β* = 1.31, *p* = 0.038) and being unmarried (*β* = 1.54, *p* = 0.038) were significantly associated with higher depression scores, whereas greater subjective support (*β* = −0.24, *p* = 0.002) and higher support utilization (*β* = −0.61, *p* = 0.025) were associated with lower depression scores. A longer duration of chronic disease was modestly linked to reduced anxiety levels (*β* = −1.05, *p* = 0.049).

Doubly robust (DR) regression analyses, combining IPTW with covariate adjustment, were conducted to verify the robustness of the associations between financial toxicity and psychological outcomes (Tables 7, 8). The findings were consistent with those of the IPTW-only models. Compared with the no financial toxicity group, participants in the moderate FT group exhibited significantly higher scores on both PHQ-9 (*β* = 11.48, *p* < 0.001) and GAD-7 (*β* = 8.31, *p* < 0.001). Those in the mild FT group also showed elevated levels of depression (*β* = 4.08, *p* < 0.001) and anxiety (*β* = 1.72, *p* = 0.009).

**Table 7:** Doubly robust weighted regression results for depression.

Variable	Estimate	Std. Error	t value	*p* value
(Intercept)	9.23	3.31	2.79	0.0055
Moderate FT (vs. No FT)	11.48	1.08	10.63	<0.001
Mild FT (vs. No FT)	4.08	0.66	6.15	<0.001
Age	1.20	0.62	1.94	0.053
Residence	−1.02	0.52	−1.96	0.050
Education	0.69	0.63	1.10	0.272
Marital status	1.50	0.72	2.08	0.039
Occupation	0.11	0.19	0.58	0.564
Insurance type	0.59	0.55	1.07	0.288
Monthly income	0.39	0.57	0.68	0.498
Disease duration	−1.11	0.52	−2.13	0.036
Relapse frequency	0.66	0.55	1.20	0.233
Subjective support	−0.23	0.08	−2.94	0.004
Support utilization	−0.58	0.27	−2.14	0.035
Avoidance dimension	−0.19	0.14	−1.43	0.154

The doubly robust model combines inverse probability of treatment weighting (IPTW) based on CBPS with covariate adjustment. Weights were trimmed at 5% and capped at 10. The outcome was PHQ-9 depression score, and the reference group was participants with no financial toxicity.

**Table 8:** Doubly robust weighted regression results for anxiety.

Variable	Estimate	Std. Error	t value	*p* value
(Intercept)	5.07	3.21	1.58	0.116
Moderate FT (vs. No FT)	8.31	1.11	7.48	<0.001
Mild FT (vs. No FT)	1.72	0.65	2.64	0.009
Age	0.94	0.67	1.41	0.159
Residence	−0.42	0.49	−0.86	0.392
Education	0.71	0.64	1.11	0.270
Marital status	0.89	0.66	1.36	0.175
Occupation	0.06	0.16	0.38	0.706
Insurance type	0.15	0.49	0.31	0.755
Monthly income	0.62	0.44	1.41	0.161
Disease duration	−1.04	0.53	−1.95	0.053
Relapse frequency	0.83	0.49	1.69	0.092
Subjective support	−0.05	0.06	−0.83	0.407
Support utilization	−0.60	0.33	−1.84	0.067
Avoidance dimension	−0.12	0.12	−1.05	0.294

This doubly robust model includes both inverse probability of treatment weighting (IPTW) and covariate adjustment. Weights were estimated via covariate balancing propensity scores (CBPS), trimmed at 5%, and capped at 10. The outcome was GAD-7 anxiety score, with the no financial toxicity group serving as the reference.

Depression was further associated with older age (*β* = 1.28, *p* = 0.046), being unmarried (*β* = 1.42, *p* = 0.045), lower subjective support (*β* = −0.22, *p* = 0.004), and reduced support utilization (*β* = −0.54, *p* = 0.035). In the anxiety model, no covariates reached statistical significance, although longer disease duration and support utilization showed marginal associations (both *p* < 0.1). These results reinforce the robustness of the observed effects of financial toxicity on psychological distress after accounting for potential residual confounding.

## Discussion

In this study, LPA identified three distinct profiles of FT among patients with cardiovascular non-cancer chronic comorbidities. The model fit indices supported the validity of this classification and highlighted substantial individual differences in FT levels. Notably, 74.8% of participants experienced mild or above FT, underscoring the need for greater attention to the multidimensional economic burden imposed on patients and their families.

Multivariate logistic regression analysis revealed several factors associated with FT profile membership. Patients aged 18–45 were more likely to belong to the no FT group, contrasting with Jing’s findings (Jing et al., 2020). The discrepancy may be attributed to the higher employment rates, more diverse income sources, and generally lower medical demands (mainly basic treatment) among younger patients with non-cancer chronic diseases. Urban residents were also more likely to fall into the no FT group, possibly due to better healthcare access, broader insurance coverage, and convenience. In contrast, rural patients often face additional time and transportation costs when seeking care. Patients without spouses were more likely to experience moderate FT, consistent with the findings of Aviki et al. (2022). This may be related to the absence of family support, which increases both psychological stress and the burden of out-of-pocket expenses. The lack of caregiver assistance may also lead to higher ancillary costs (e.g., for nurse aides or escorts). Patients with junior or senior high school education were more likely in the no FT group, possibly due to their basic health literacy and greater tendency to select cost-effective treatment options, such as prioritizing medications covered by health insurance. Retirees were more likely to experience mild FT, differing from Su’s findings (Su et al., 2020) in cancer patients. This discrepancy may be attributed to disease characteristics: unlike cancer, non-cancer chronic diseases typically do not require intensive, high-cost treatment over short periods. However, the long-term medication burden and management of complications may still strain the fixed pension income of retirees.

Interestingly, patients with a per capita monthly household income ≤3,000 RMB were more likely to fall into the no FT group, while those with incomes ≥5,000 RMB or who self-financed their care were more likely to experience moderate FT. This contrasts with Li’s findings (Li et al., 2024), and may be explained by a higher likelihood among high-income patients to choose expensive, non-reimbursable treatments (e.g., imported stents or private hospital services), leading to increased out-of-pocket expenses. Prior research has confirmed that high out-of-pocket costs are an independent risk factor for FT (Parikh et al., 2019). The analysis revealed that patients with two chronic conditions were more likely to experience mild or moderate FT, consistent with previous findings (Chen et al., 2018). Compared to those with a single condition, patients with two or three comorbidities incur significantly higher costs—on average, 3.4 times more—largely due to the need for multispecialty care and combined medication regimens (e.g., cardiac rehabilitation alongside diabetes management). However, the addition of more than three chronic diseases did not proportionally increase costs, possibly due to insurance caps or standardized pathways controlling costs in some regions, highlighting the need for optimized comorbidity management. Shorter disease duration (≤1 year) correlated with the no FT group, as early standardized management reduces long-term complication risks and unplanned hospitalizations (Handelsman et al., 2023). Similarly, patients with ≤1 annual relapse were more likely to have only mild FT, emphasizing the role of regular follow-up and self-monitoring in preventing costly exacerbations. Counterintuitively, higher levels of subjective support and support utilization were associated with mild to moderate FT, potentially indicating overreliance weakening self-management and coping abilities. Additionally, patients with an avoidance-oriented coping style were more likely to fall into the moderate FT group. This may be due to delayed care or self-initiated medication reduction, which can lead to disease progression and increased treatment costs.

Beyond the factors associated with latent profile classification, this study further confirmed the psychological implications of FT through propensity score–based weighted regression. After adjusting for baseline differences via covariate balancing propensity scores, both mild and moderate FT were significantly associated with increased GAD-7 and PHQ-9 scores, with the moderate group showing the greatest burden. These findings are in line with recent evidence highlighting FT as a key predictor of psychological distress among patients with chronic diseases, especially those with cardiovascular comorbidities. For example, recent studies have emphasized that FT contributes to delayed care, non-adherence to medication, and increased psychological distress in heart failure patients (Sharma et al., 2024). A study using logistic regression found that anxiety and depression in patients with coronary heart disease and subclinical hypothyroidism were significantly influenced by financial strain, even after adjusting for clinical parameters (Meng et al., 2024). Furthermore, the burden of out-of-pocket costs and the threat of medical bankruptcy have been increasingly recognized as underappreciated drivers of poor mental health and health outcomes in cardiovascular populations (Khera et al., 2020).

The mechanisms driving FT and its consequences are multifaceted. Objectively, long-term disease management often requires multiple medications (e.g., anticoagulants, lipid-lowering agents, glucose-lowering therapies) to maintain clinical stability. The cumulative costs of ongoing treatment and repeated hospitalizations—exacerbated by delayed care and complications such as myocardial remodeling, peripheral vascular disease, and renal impairment—represent a significant financial burden. Subjectively, frequent medical visits disrupt daily life and contribute to psychological distress, including anxiety and depression, in both patients and caregivers. These emotional strains are further intensified by the associated financial losses, creating a bidirectional cycle of psychosocial and economic stress (Felez-Nobrega et al., 2022). To reduce healthcare costs, economically disadvantaged patients often resort to suboptimal coping strategies, such as reducing the frequency of follow-up visits or altering medication dosages (Osborn et al., 2017), which may trigger a vicious cycle of “financial burden – reduced treatment efficacy – increased costs.”

Our findings suggest integrating FT screening into clinical workflows could improve patient care. Using tools like the COST-PROM scale during patient intake or regular visits helps identify those at high risk for financial distress. Early identification allows for targeted interventions, such as financial counseling and assistance with insurance navigation. To mitigate FT’s dual impact on mental and economic well-being, a two-pronged intervention model integrating subjective initiative and external support is recommended:

Dynamic assessment & tailored care: a dynamic FT assessment mechanism should be incorporated into clinical decision-making. Incorporating FT screening tools, such as the COST-PROM scale, during patient intake or annual assessments can help healthcare providers identify patients who may be experiencing significant financial strain. Treatment plans must be tailored based on patients’ financial capacity, insurance coverage, and community resources. Cost–benefit considerations should be integrated into treatment selection (Warraich et al., 2020), employing stepped dosing strategies to balance efficacy and economic feasibility.

Financial & resource navigation: leveraging health insurance benefits, charitable aid, and outpatient chronic disease programs can minimize out-of-pocket expenses. Promoting alignment between basic and supplemental insurance schemes can help high-income patients balance quality and cost-effectiveness. Future directions include developing dynamic financial navigation systems for household planning and establishing community-based mutual aid funds.

Support systems & capacity building: establish social support networks for vulnerable groups (e.g., unmarried, living alone). Integrate community resources for targeted financial assistance. Strengthen primary care capacity (e.g., via digital remote monitoring platforms for alerts/reminders) to reduce avoidable hospitalizations. Medical institutions should promote patient-centered health education and peer support to enhance self-management. Community services should offer accessible psychological counseling and financial navigation resources to build resilience and manage stress.

Integrated psychosocial care: combining FT screening with mental health assessments (e.g., GAD-7, PHQ-9) provides a comprehensive approach. Patients with significant FT and distress should be referred to social workers or mental health professionals.

This study identified distinct FT profiles and their correlates in patients with cardiovascular non-cancer chronic comorbidities, confirming FT’s significant association with psychological distress. It underscores FT as not merely a reflection of socioeconomic disadvantage but potentially an independent determinant of psychological well-being. By providing a theoretical foundation for targeted interventions, this research aims to alleviate financial burden and improve long-term health outcomes in this vulnerable population. Implementing the proposed screening and multifaceted support strategies is crucial for achieving equitable and sustainable chronic disease management.

However, due to the cross-sectional design of this study, the temporal sequence between FT and psychological distress cannot be established, and causal interpretations should be made with caution. In addition, the use of self-reported psychological measures may introduce recall and social desirability biases, potentially affecting the accuracy of symptom assessment. These limitations should be considered when interpreting the findings and especially when deriving clinical or policy recommendations. Future longitudinal studies with objective or clinician-rated assessments are warranted to validate these associations.

### Limitations

This study has several limitations. First, the sample was primarily drawn from a single tertiary hospital in Wuhan, which may limit the generalizability of findings, especially to rural residents or those in primary or secondary care settings. Future studies should adopt multi-center, population-based sampling to improve external validity. Second, as a cross-sectional study, it cannot capture changes in financial toxicity over time. Future research should expand the study population and conduct longitudinal follow-up to better understand the progression of financial toxicity and provide a stronger foundation for evaluation and intervention strategies. Third, LPA was based solely on the 11 items of the COST-PROM scale and did not incorporate other potentially important variables such as patients’ economic status and healthcare utilization. These factors may have a significant impact on financial toxicity but were not included due to study design constraints. Future studies are encouraged to integrate multidimensional data—such as economic, medical, psychological, and social support factors—into latent profile modeling to improve the accuracy and applicability of classification results. Fourth, although the study employed CBPS, IPTW, and DR estimation to adjust for confounding, the possibility of residual confounding due to unmeasured variables cannot be completely excluded. These statistical approaches strengthen causal inference but do not establish definitive causality. Future studies may benefit from incorporating prospective designs or quasi-experimental methods to more rigorously assess causal pathways.

## Conclusion

This study identified three latent profiles of financial toxicity among patients with cardiovascular non-cancer chronic diseases, with most experiencing mild to moderate financial burden. Factors such as age, income, education, disease characteristics, and coping style were associated with these profiles. Moreover, after adjusting for confounders using covariate balancing propensity scores and doubly robust estimation, financial toxicity remained significantly associated with higher levels of anxiety and depression, suggesting an independent psychological impact. These findings highlight the need for early identification and multidimensional intervention strategies. Targeted support, proactive management, and improved integration of medical and social resources may help alleviate financial strain and enhance both mental health and clinical outcomes.

## Data Availability

The raw data supporting the conclusions of this article will be made available by the authors, without undue reservation.
